# Phylogeography and Population Structure of *Glossina fuscipes fuscipes* in Uganda: Implications for Control of Tsetse

**DOI:** 10.1371/journal.pntd.0000636

**Published:** 2010-03-16

**Authors:** Jon S. Beadell, Chaz Hyseni, Patrick P. Abila, Rogers Azabo, John C. K. Enyaru, Johnson O. Ouma, Yassir O. Mohammed, Loyce M. Okedi, Serap Aksoy, Adalgisa Caccone

**Affiliations:** 1 Department of Ecology and Evolutionary Biology, Yale University, New Haven, Connecticut, United States of America; 2 National Livestock Resources Research Institute, Tororo, Uganda; 3 Department of Biochemistry, Faculty of Science, Makerere University, Kampala, Uganda; 4 Trypanosomiasis Research Centre, Kenya Agricultural Research Institute, Kikuyu, Kenya; 5 Central Veterinary Research Laboratories, Animal Resources Research Corporation, Khartoum, Sudan; 6 Department of Epidemiology and Public Health, Yale University, New Haven, Connecticut, United States of America; Foundation for Innovative New Diagnostics (FIND), Switzerland

## Abstract

**Background:**

*Glossina fuscipes fuscipes*, a riverine species of tsetse, is the main vector of both human and animal trypanosomiasis in Uganda. Successful implementation of vector control will require establishing an appropriate geographical scale for these activities. Population genetics can help to resolve this issue by characterizing the extent of linkage among apparently isolated groups of tsetse.

**Methodology/Principal Findings:**

We conducted genetic analyses on mitochondrial and microsatellite data accumulated from approximately 1000 individual tsetse captured in Uganda and neighboring regions of Kenya and Sudan. Phylogeographic analyses suggested that the largest scale genetic structure in *G. f. fuscipes* arose from an historical event that divided two divergent mitochondrial lineages. These lineages are currently partitioned to northern and southern Uganda and co-occur only in a narrow zone of contact extending across central Uganda. Bayesian assignment tests, which provided evidence for admixture between northern and southern flies at the zone of contact and evidence for northerly gene flow across the zone of contact, indicated that this structure may be impermanent. On the other hand, microsatellite structure within the southern lineage indicated that gene flow is currently limited between populations in western and southeastern Uganda. Within regions, the average F_ST_ between populations separated by less than 100 km was less than ∼0.1. Significant tests of isolation by distance suggested that gene flow is ongoing between neighboring populations and that island populations are not uniformly more isolated than mainland populations.

**Conclusions/Significance:**

Despite the presence of population structure arising from historical colonization events, our results have revealed strong signals of current gene flow within regions that should be accounted for when planning tsetse control in Uganda. Populations in southeastern Uganda appeared to receive little gene flow from populations in western or northern Uganda, supporting the feasibility of area wide control in the Lake Victoria region by the Pan African Tsetse and Trypanosomiasis Eradication Campaign.

## Author Summary

*Glossina fuscipes fuscipes* is the most common species of tsetse in Uganda, where it transmits human sleeping sickness and nagana, a related disease of cattle. A consortium of African countries dedicated to controlling these diseases is poised to begin area wide control of tsetse, but a critical question remains: What is the most appropriate geographical scale for these activities? To address this question, we used population genetics to determine the extent of linkage between populations of tsetse confined to discrete patches of riverine habitat. Our results suggest that Uganda was colonized by two distinct lineages of *G. f. fuscipes*, which now co-occur only in a narrow band across central Uganda. Evidence for interbreeding at the zone of contact and movement of genes from the south to the north suggest that this historical genetic structure may dissolve in the future. At smaller scales, we have demonstrated that exchange of genes among neighboring populations via dispersal is at equilibrium with the differentiating force of genetic drift. Our results highlight the need for investment in vector control programs that account for the linkage observed among tsetse populations. Given its genetic isolation and its location at the far edge of *G. fuscipes*' range, the Lake Victoria region appears to be an appropriate target for area wide control.

## Introduction

Human African Trypanosomiasis, or sleeping sickness, is a vector-borne disease that kills thousands of people each year in sub-Saharan Africa [1]. Nagana, a related disease of livestock, can also be fatal and is a major impediment to agricultural development. Both diseases are caused by parasitic trypanosomes transmitted by tsetse flies. No vaccines exist to prevent the disease and drugs currently available to treat sleeping sickness in humans are expensive, can cause severe side effects and are difficult to administer in remote villages. Therefore, controlling the tsetse vector, via methods involving habitat elimination, trapping, insecticide-treated targets, aerial or ground spraying, insecticide-treated cattle, or the release of sterile or transgenic insects, may represent an effective alternative for controlling the disease [2]–[6]. In 2001, the Pan-African Tsetse and Trypanosomiasis Eradication Campaign (PATTEC) was formed with the goal of identifying discrete zones of infestation that could be systematically exterminated, one by one, using area-wide methods [6],[7]. Geographic features such as mountains and bodies of water were originally proposed as useful boundaries to delineate isolated target populations, however, biologically-relevant boundaries may be better defined by molecular techniques that explicitly account for the degree of interaction between neighboring groups of flies.

The application of population genetic methods to disease vectors offers insights into facets of ecology, reproduction, dispersal patterns, population dynamics and genetic diversity that can have direct relevance for guiding control programs. At deeper timescales, population genetics can elucidate the historical processes that have influenced the current distribution of vector populations. Over more recent timescales, population genetics can delineate isolated vector populations that may be appropriate targets for local eradication and can identify dispersal routes that must be disrupted to prevent reinfestation of previously cleared zones. Characterization of barriers to gene flow is particularly important in planning the release of genetically modified or sterile vectors where the scale of success will depend on the scale at which the released vectors mate with wild counterparts [8],[9]. Beyond this, estimates of genetic diversity and effective size can provide insight into the relative importance of drift or selection in driving important phenotypes such as insecticide resistance and vector competence. Finally, if established early enough, ongoing genetic monitoring can help to characterize the effectiveness of control efforts (i.e., by estimating the reduction in effective population sizes resulting from control) and can help to identify the cause of any failures (i.e., whether flies have recolonized from relict populations within controlled areas or from neighboring populations outside of control areas).

**Figure 1:**
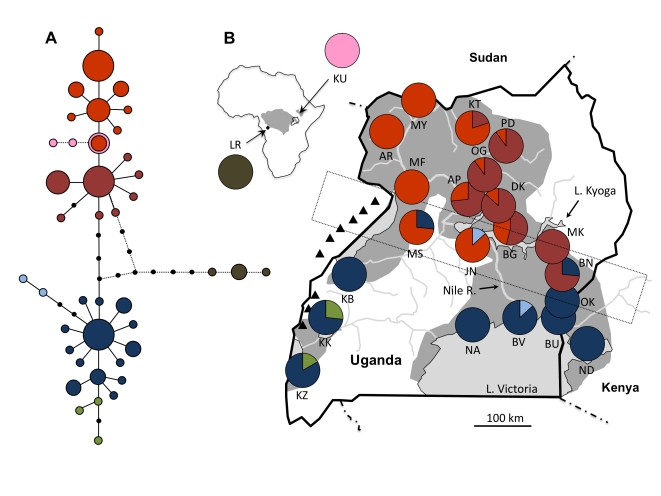
Parsimony network of mitochondrial haplotypes recovered from *G. f. fuscipes* and their distribution in Uganda, Kenya and Sudan. In the network (A), haplotypes are represented by circles, sized proportional to the frequency with which those haplotypes were recovered and shaded to represent clades composed of equivalent mutational steps. Black dots represent unsampled haplotypes. Haplotypes shaded in pink were obtained from a disjunct population of *G. f. fuscipes* in Sudan. Haplotypes shaded in brown were obtained from a population of *G. f. quanzensis* from the Democratic Republic of the Congo. Pie charts on the map (B) indicate the frequency with which clades of related haplotypes (identified in A) occurred in each population. Background shading indicates the predicted range of *G. f. fuscipes* in the region and in Africa (inset; [67]). Triangles mark the location of the Rwenzori and Blue Mountains. The dotted rectangle indicates a zone of contact between northern and southern mitochondrial haplotypes.

In Uganda, the primary vector of both human and animal forms of trypanosomiasis is *Glossina fuscipes fuscipes* Newstead 1910, which occurs in a peninsular distribution terminating around the shores of Lake Victoria at the far eastern edge of its range (see Figure 1). Given the possibility of severing this peninsula from the remainder of *fuscipes*' range in central Africa, the Lake Victoria region has been proposed as a potentially suitable target for area-wide control [6]. Although the severe HAT epidemics of the 20th century have largely subsided [10], the acute form of the disease (*Trypanosoma brucei rhodesiense*) has recently extended its range from a historical focus along the shores of Lake Victoria to several foci extending north into central Uganda [11]. This has raised concern that its distribution will eventually overlap with that of the chronic form of the disease (*T. b. gambiense*) found in northwestern Uganda, complicating both the diagnosis and treatment of the disease.

Genetic work by Abila et al. [12] did not find any evidence to suggest that the distribution of trypanosome forms in Uganda is constrained by the distribution of tsetse genotypes. However, in support of proposed vector control activities, this research highlighted a major discontinuity in genotypes between tsetse in central/northern Uganda and those found in the Lake Victoria region to the south. Lake Kyoga, in central Uganda, was identified as the main factor behind this genetic structure, but limited sampling prevented these authors from identifying the actual zone of contact between northern and southern populations and from assessing the level of admixture between these populations in locations where they may coexist. Nuclear data confirmed the genetic discontinuity observed in mtDNA, but the use of only five microsatellite loci prevented finer-scale resolution of population structure.

In Uganda, *G. f. fuscipes* is restricted to discrete patches of riverine and lacustrine habitat distributed among large, uninhabitable tracts of pastures and agricultural fields. The extent to which these apparently discrete populations are actually linked by dispersal is unknown, but preliminary work in southeastern Uganda has suggested that even flies from the same watershed may share little mitochondrial variation [13]. Fully understanding the population structure of tsetse in Uganda at local scales will be important for defining the scale on which vector control is likely to be most effective and, eventually, for elucidating the extent to which local interactions between tsetse and trypanosomes may be influencing disease epidemiology. Therefore, we undertook an in depth population genetic study on a geographically comprehensive scale in order to resolve the hierarchical structure of *G. f. fuscipes* in Uganda.

## Materials and Methods

### Field Sampling

Tsetse were caught in biconical traps [14] set amongst low bushes or in gallery forests along rivers, lakes, seeps or other semi-permanent water sources. In general, traps from a single site were placed within a 1–5 km^2^ area. We collected flies from 21 sites across Uganda as well as single sites in Ndere Island, Kenya (ND) and Kurmuk, Sudan (KU; see Figure 1). S. Torr kindly provided specimens of *G. f. quanzensis* from the Lukaya River in the Democratic Republic of Congo (LR). All samples were collected between 2006 and 2009, except for flies from Kigezi (KZ) and Kitgum (KT), which had been collected in 1974 and 1997, respectively, and were obtained from a museum collection stored at the Coordinating Office for the Control of Trypanosomiasis in Uganda.

### Genetic Methods

Flies caught between 2006 and 2009 were extracted using DNeasy kits (Qiagen, Valencia, CA) following the manufacturer's protocols. Museum specimens were extracted using the DNA-IQ system (Promega, Madison, WI) in a dedicated ancient DNA laboratory in order to prevent contamination with more abundant sources of modern DNA.

Attempts to amplify fragments of cytochrome oxidase I (COI) and cytochrome b (cytb) genes using primers designed previously for *G. fuscipes* [12] yielded multiple peaks in sequence chromatograms (K. Dion pers. comm.; unpubl. data), suggesting the presence of nuclear copies. Therefore, we designed new primers COIF1 (5′ – CCT CAA CAC TTT TTA GGT TTA G – 3′) and COIIR1 (5′ – GGT TCT CTA ATT TCA TCA AGT A – 3′), which consistently amplified just a single sequence. To help insure that this sequence originated from the mitochondrial genome and not a nuclear copy (NUMT), we compared sequences obtained with COIF1/COIIR1 (570 bp) to sequences obtained from the amplification of an ∼1.7 kb fragment (using C1-J-1751/C2-N-3389; [15]), which is larger than most NUMTs found in eukaryotic genomes [16]. In all cases, sequences matched identically. We amplified COIF1/COIIR1 in a 30 µl PCR reaction employing 1X buffer (Applied Biosystems, Foster City, CA), 0.2 mM each dNTP, 0.6 µM primers, 2.0 mM MgCl_2_, 0.4 mg/mL BSA and 0.6 U AmpliTaq Gold (Applied Biosystems) using 50 cycles and an annealing temperature of 51°C. All sequences have been deposited in GenBank (Table S1).

The majority of published microsatellite loci that had been developed for *Glossina fuscipes* [12],[17] could not be utilized due to apparent X-linkage (B03, D12, C107, D6, D109, A6, A9, A112), amplification problems, or scoring difficulty (unpubl. data). Therefore, we adapted 12 loci that had previously been developed for *G. pallidipes* [18], *G. palpalis* [19] and *G. morsitans* [20] for use in *G. fuscipes*. In addition, we developed one new locus (C07b) from the *G. fuscipes* microsatellite library used by Brown et al. [17]. Amplifications were performed using a touchdown PCR (10 cycles of annealing at progressively lower temperatures from 60°C to 51°C followed by 35 cycles at 50°C) in 12.5 µl reaction volumes employing 1X buffer, 0.2 mM dNTPs, 2.0 mM MgCl_2_, 0.2 mg/mL BSA and 0.5 U AmpliTaq Gold. Flourescently-labeled primers were included at a final concentration of 0.4 µM. When using the m13 amplification system [21], the m13–tagged forward primer was included at a final concentration of 0.05 µM while the flourescently-labeled m13 primer and reverse primers were used at a concentration of 0.4 µM. Primer sequences and original sources for all loci are summarized in Table S2. Microsatellite data were collected on an ABI 3730 and alleles were scored using the program GeneMarker (SoftGenetics, State College, PA).

### Descriptive Statistics and Marker Validation

For mtDNA, we calculated haplotype diversity (H_d_) and nucleotide diversity (π) using the program DnaSP v5 [22]. For microsatellites, we tested for deviations from Hardy Weinberg equilibrium (HWE) and for linkage disequilibrium (LD) among all pairs of loci across all populations using the program Genepop v4.0 [23]. Markov chain parameters were set at 10,000 dememorizations, 1000 batches, 10,000 iterations per batch for HWE and 100,000 dememorizations, 1000 batches, 10,000 iterations per batch for LD. We used the program FSTAT v2.9.3.2 [24] to calculate allelic richness as well as observed (H_o_) and expected (H_e_) heterozygosity for each population.

### Phylogeographic History

We evaluated evolutionary relationships among mitochondrial lineages using a parsimony network generated by the program TCS version 1.21 [25]. Next, we employed mismatch distributions [26] to help visualize signatures of population growth within two distinct clades of haplotypes identified in the parsimony network. We calculated the raggedness statistic (r) as an indicator of the extent to which the distribution followed the smooth unimodal curve expected to result from a growing population. We also tested for deviations from neutral expectations using Fu's F_s_ [27] and Ramos-Onsins' R_2_ [28] statistics. These have been demonstrated to be the most powerful tests available for detecting population growth [28]. Fu's F_s_ assesses the probability of the observed haplotype distribution occurring under conditions of neutrality and tends to be negative when there is an excess of recent mutations due to population growth, genetic hitchhiking, or background selection. R_2_, on the other hand, assesses the difference between the number of singleton mutations observed in a population and the average number of nucleotide differences in that population. The increased number of singleton mutations occurring after a recent episode of severe population growth should result in low values of R_2_. Tests of demographic expansion were conducted using DnaSP and significance was evaluated by comparing the observed statistics to a distribution of values generated with 5000 coalescent simulations.

### Population Structure and Gene Flow

We calculated pairwise F_ST_ values for both mitochondrial and microsatellite data as a basic index of population differentiation. F_ST_ reflects the proportion of variance in haplotype or allele frequencies that is attributable to differentiation between populations relative to the total variance observed across all populations (see [29] for a review of population genetic definitions). We calculated F_ST_ using the program Arlequin v3.1 [30] and tested for significant differences using 10,000 permutations. Significance was assessed using the sequential Bonferroni method.

We employed microsatellite data and a Bayesian clustering method implemented in the program STRUCTURE v2.2 [31] to estimate the number (K) of clusters (metapopulations) present and to assign individual tsetse to these metapopulations without using prior information on sampling locality. The latter feature allowed for identification of individuals exhibiting recent ancestry from a metapopulation other than the one from which it was sampled, thereby polarizing the directionality of putative dispersal events. To identify the most likely K, we conducted 3 independent runs for each K from K = 1 to 15 assuming an admixture model with uncorrelated allele frequencies. We used a burn-in of 50,000 and replication values of 250,000. We used the guidelines outlined by Pritchard et al. [31] and the second order rate of change in the likelihood distribution (ΔK; [32]) to identify a K that was most consistent with the data. For final assignment of individuals to the likeliest number of metapopulations, we reran STRUCTURE with a burn-in of 100,000 steps and replication value of 500,000. We confirmed the STRUCTURE results using the program Geneland [33] using a nonspatial model and 500,000 steps for K = 1 to 15.

We estimated the proportion of variance explained by populations (sampling sites) within metapopulations and also the variance explained by metapopulations (identified at higher K) within larger metapopulations (identified at lower K) using the program HIERFSTAT [34]. Significance of the differentiation was tested by comparing an observed G-statistic to that obtained with 1000 randomized data sets.

In order to further explore the hierarchical relationships among populations, we generated a neighbor-joining tree based on Cavalli-Sforza chord distances. We generated genetic distances using the program MSA v4.05 [35] and produced the tree using the program NEIGHBOR in Phylip 3.68. Robustness of groupings was evaluated by using MSA v 4.05 to generate 1000 bootstrap replicates and using the program CONSENSE in Phylip 3.68 to identify the proportion of replicates exhibiting identical groupings.

To assess the relative influences of drift and gene flow, we tested for a positive association between genetic distance (F_ST_/(1- F_ST_); [36]) and geographic distance using the Isolation By Distance Web Service v 3.16 [37]. We employed a one-dimensional framework given the scale of sampling relative to the width of predicted habitat corridors. Significance of the associations was tested with a Mantel test employing 10,000 randomizations. We tested for isolation by distance (IBD) using F_ST_, a frequency based measure, as well as Φ_ST_, which also accounts for the evolutionary relatedness of haplotypes (mtDNA only). For mitochondrial data, we tested each of the two distinct groups of lineages (north and south) identified in a parsimony network, but excluded populations at the zone of contact between these two groups (MS, JN and BN; see Figure 1). For microsatellite data, we tested for IBD in the northern and southern clusters identified by STRUCTURE at K = 2. We also evaluated IBD in each of the two clusters that arose from the southern cluster (west and southeast) at K = 3. In all analyses, we excluded populations from Sudan and the DRC as well as populations represented by fewer than 10 (mtDNA) or 20 (microsatellites) individuals.

### Population Size and Stability

We performed single sample point estimates of effective population size (N_e_), the number of individuals in an ideal population (subject only to random genetic drift) that would exhibit the same distribution of allele frequencies as actually observed. We calculated N_e_ using both linkage disequilibrium (LDNE; [38]) and Bayesian (ONeSAMP; [39]) methods. For ONeSAMP, estimates of N_e_ that rise with increasing upper limits on the prior are indicative of large N_e_ values (D Tallmon pers. comm.); therefore, we explored the stability of estimates using priors of both 5,000 and 10,000 for maximum N_e_. We also evaluated all populations for evidence of a recent decline in effective population size using the program BOTTLENECK [40]. We performed the test using both the infinite allele (IAM) and two-phase (TPM) models of microsatellite evolution. We assessed the significance of the tests using Wilcoxon's test, which is the most appropriate test in cases where fewer than 20 microsatellite loci are used [40].

## Results

### Microsatellite Validation

After excluding the two least polymorphic loci (C5b and CAG29) and using the sequential Bonferroni method to account for non-independence of multiple tests, we detected no significant tests of linkage disequilibrium among the 55 remaining pairs of loci. Using the generalized binomial procedure implemented in MultiTest v.1.2 [41] to control for multiple tests, we identified two of 13 loci (D05 and Pgp17) that exhibited a significant proportion of populations with F_IS_ values significantly different than zero (Table S3). Six population-by-locus combinations yielded significant (p<0.002) deviations from Hardy Weinberg expectations after Bonferroni correction (performed by locus). Analyses performed with the program Micro-Checker [42] indicated that null alleles at loci D05 and Pgp17 were likely responsible for heterozygote deficiencies observed in these populations. Re-amplification of several homozygous individuals using less stringent conditions for both D05 and Pgp17 did not resolve the problem. We performed all major analyses with and without these loci but report only results for 13 loci since the results were consistent across both modes of analysis.

### Genetic Diversity

In Uganda and the single population from Kenya, we identified 36 unique mitochondrial haplotypes among 284 individuals for which we sequenced mitochondrial DNA. We recovered two additional haplotypes from 15 Sudan specimens and three haplotypes from 10 specimens of *G. f. quanzensis* from the DRC. Among samples of *G. f. fuscipes*, overall haplotype diversity (H_d_) was 0.906. Across individual populations, H_d_ ranged from a high of 0.778 in the Uganda population from Ogur (OG) to a low of 0 in the Kenyan population from Ndere Island (ND; Table 1).

**Table 1:** Sample sizes and genetic diversity statistics at mitochondrial and microsatellite loci for populations of *G. f. fuscipes*.

		mtDNA				Microsatellites			
Location	Code	N	# Haplotypes	H_d_	π	N	Allelic Richness c	H_o_	H_e_
**Uganda**									
Apac	AP	15	6	0.762	0.00261	49	5.5	0.573	0.624
Arua	AR	15	5	0.619	0.00127	40	6.7	0.706	0.705
Bugondo	BG	13	3	0.667	0.00373	13	-	0.574	0.570
Pallisa	BK	-	-	-	-	40	4.0	0.494	0.506
Bunghazi	BN	15	3	0.648	0.00501	40	4.5	0.504	0.553
Busime	BU	15	2	0.533	0.00094	40	4.0	0.450	0.480
Buvuma	BV	15	4	0.724	0.00247	39	4.9	0.487	0.498
Dokolo	DK	15	3	0.533	0.00147	64	4.8	0.503	0.565
Junda	JN	15	2	0.248	0.00478	40	3.8	0.439	0.445
Kabunkanga	KB	15	3	0.257	0.00470	40	5.9	0.594	0.613
Kakoga	KK	15	4	0.543	0.00120	40	5.3	0.498	0.548
Kalengera	KL	-	-	-	-	40	3.8	0.463	0.460
Kitgum a	KT	5	2	0.400	0.00140	-	-	-	-
Kigezi a	KZ	6	3	0.600	0.00269	-	-	-	-
Murchison Falls	MF	15	3	0.257	0.00047	39	6.9	0.620	0.645
Mukongoro	MK	15	2	0.533	0.00094	40	3.1	0.425	0.426
Masindi	MS	15	2	0.419	0.00735	40	4.1	0.563	0.567
Moyo	MY	15	3	0.59	0.00117	41	6.7	0.617	0.632
Nkumba	NA	15	2	0.514	0.00180	36	2.9	0.478	0.449
Ndere (Kenya) b	ND	15	1	0	0	40	2.3	0.302	0.299
Ogur	OG	10	6	0.778	0.00246	37	6.1	0.612	0.619
Okame	OK	15	3	0.362	0.00067	40	3.9	0.487	0.498
Osuguro	OS	-	-	-	-	32	4.3	0.497	0.523
Pader	PD	10	2	0.200	0.00070	13	-	0.645	0.625
North		163	17	0.830	0.00336				
South		121	19	0.786	0.00258				
Uganda Total		284	36	0.906	0.00862				
**Sudan**									
Kurmuk	KU	15	3	0.362	0.00084	22	3.8	0.417	0.414
**Dem. Rep. Congo**									
Lukaya River	LR	10	3	0.511	0.00097	20	5.4	0.600	0.621

a Museum samples.

b Given its proximity to Uganda, the population from the Kenyan island of Ndere was grouped with Ugandan populations for all diversity estimates.

c Allelic richness was calculated for populations with a minimum of 20 individuals sampled.

**Figure 2:**
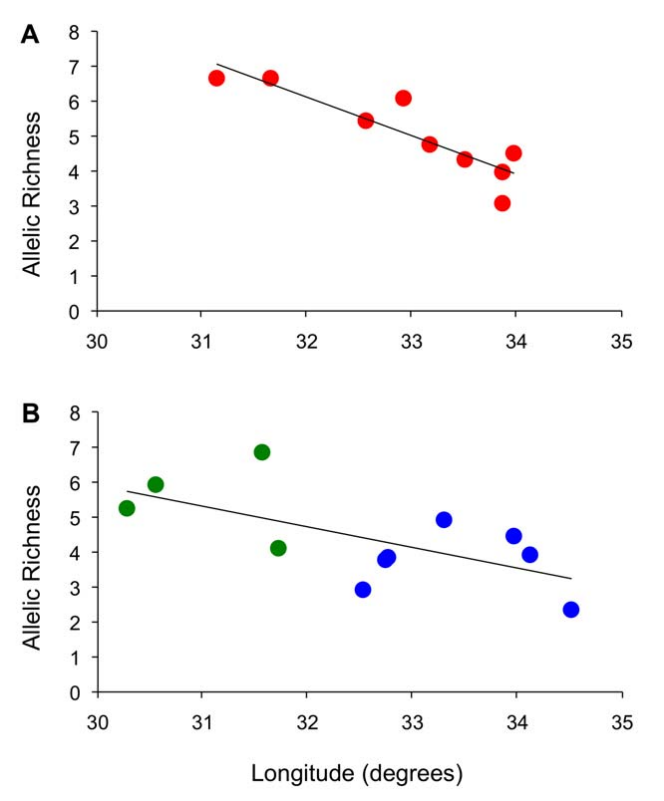
Plots of allelic richness versus longitude. Populations in northern (A) and (B) southern Uganda exhibited significant declines in allelic richness from west to east. No significant trends were apparent when the southern populations were partitioned into the western (green) and southeastern (blue) clusters identified by STRUCTURE (K = 3).

We observed high levels of microsatellite diversity in flies sampled in western Uganda at Arua (AR), Murchison Falls (MF) and Moyo (MY; Table 1, Table S4). A regression of microsatellite allelic richness on longitude (Figure 2) revealed a significant decline in diversity from west to east across both northern (R^2^ = 0.80, df = 7, p = 0.001) and southern populations (R^2^ = 0.41, df = 9, p = 0.017), however the latter trend was not evident when the southern group was further parsed into western and southeastern groups.

Island populations did not exhibit uniformly low diversity. As observed with mitochondrial diversity, Ndere Island flies exhibited the lowest levels of microsatellite variation. Flies from Buvuma Island (BV), on the other hand, exhibited allelic richness and heterozygosity levels on par with neighboring populations from the mainland (Table 1).

### Phylogeographic History

Mitochondrial haplotypes obtained from flies collected across Uganda and Sudan fell into five clades (each consisting of lineages separated by at most two mutations) that were nested within two major groups based on a parsimony network (Figure 1A). These two groups of haplotypes (henceforth north and south) were equally divergent from *G. f. quanzensis* and were perfectly partitioned in geographical space (Figure 1B). The two major groups co-occurred only at three sites (Masindi (MS), Junda (JN), Bunghazi (BN)) lying in a narrow band across central Uganda. We estimated the approximate divergence time between northern and southern groups based on the genetic distance between these two groups, accounting for ancestral polymorphism using a correction for molecular diversity observed within each group. We used the formula D_xy_ = D – 0.5*(D_x_+D_y_) where D is the mean nucleotide diversity observed between the two clades, and D_x_ and D_y_ are the mean nucleotide diversities within group x and group y [43]. Using a *Drosophila* mitochondrial mutation rate of 5.7×10^−8^ per silent site per year [44], we estimated the time of divergence of the two groups to be about 340,000 years ago.

**Figure 3:**
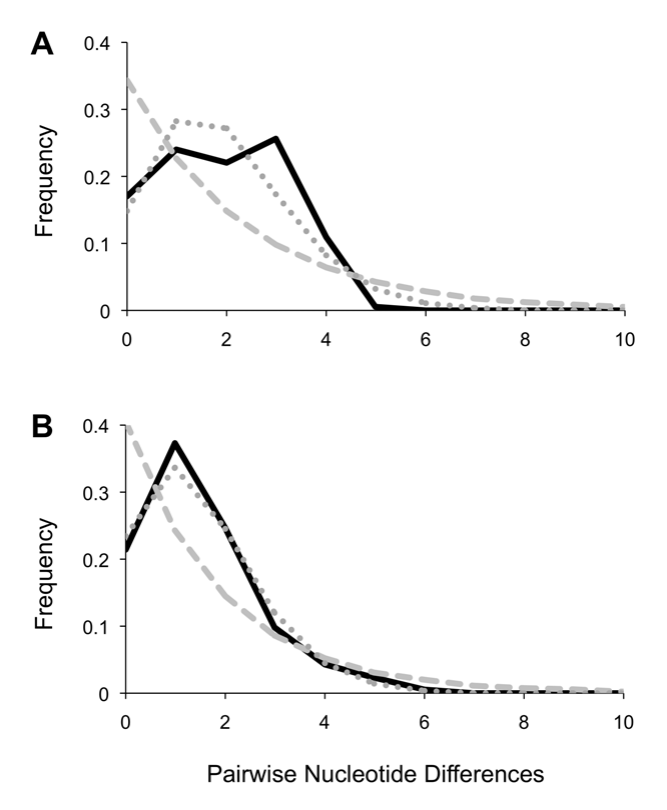
Mismatch distributions. Plots indicate the frequency of nucleotide differences observed (in black) across pairwise comparisons of mitochondrial sequences derived from *G. f. fuscipes* in northern (A) and southern (B) Uganda. For comparison, expected distributions are shown for populations exhibiting constant size (dashed grey) and exponential growth (dotted grey).

Given that northern and southern flies appear to have evolved independently for an extended period of time, we performed tests of neutrality/population growth for each group separately. Mismatch tests revealed curves typical of expanding populations, particularly in the south (Figure 3). This was confirmed by raggedness values that failed to reject the null hypothesis of exponential growth in both the north (r = 0.0389, p = 0.09) and the south (r = 0.0673, p = 0.20). A hypothesis of exponential growth was also supported by Fu's F_s_ in both the north (F_s_ = −5.5, p = 0.03) and the south (F_s_ = −11.8, p = 0); however, Ramos-Onsins' R_2_ was only marginally significant in the south (R_2_ = 0.038, p = 0.06).

### Population Structure and Gene Flow

**Figure 4:**
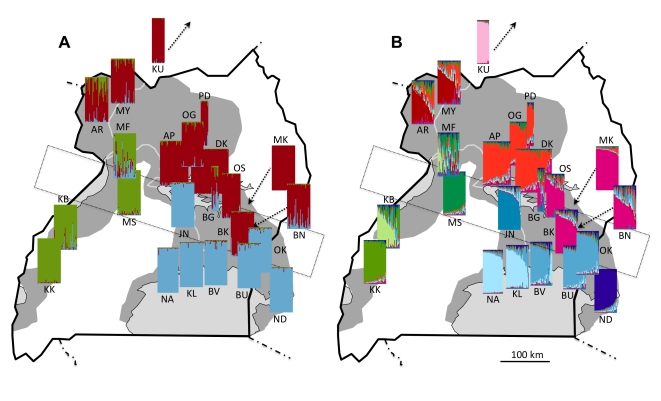
Population structure of *G. f. fuscipes* in Uganda based on a Bayesian assignment test. The probability that an individual belongs to one of 3 (A) or 11 (B) hypothetical metapopulations (identified by color), is indicated by the color composition of the individual vertical bars comprising each plot. The dotted rectangle indicates a zone of contact between northern and southern mitochondrial haplotypes.

**Figure 5:**
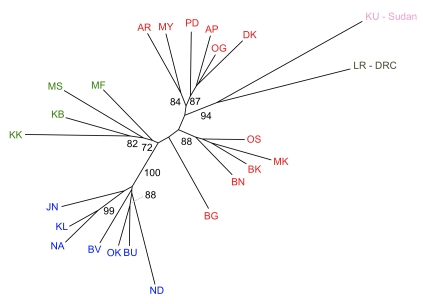
Neighbor-joining tree of Cavalli-Sforza chord distances indicating hierarchical relationships among populations. Numbers indicate bootstrap support values above 70%.

At the finest scale, pairwise F_ST_ values calculated from microsatellite data revealed significant differentiation among all sampled localities, with the sole exception of Ogur (OG) and Pader (PD; Table S5). However, Bayesian analysis of population structure evaluated with Evanno's criterion ΔK, indicated that our data were most consistent with the presence of two metapopulations. Because this division simply reflected the strong north/south division previously observed in mitochondrial haplotypes, we explored further partitions. The next highest value of ΔK supported three metapopulations (Figure 4A), in which individuals from the four western populations (Murchison Falls (MF), Masindi (MS), Kabunkanga (KB), Kakoga (KK)) split from individuals in the southeast. Hierarchical partitioning of variance yielded an F_ST_ of 0.10 (p = 0.001) for the effect of population measured within these three metapopulations. Beyond this level of partitioning, individuals continued to exhibit geographically relevant clustering up to K = 11 (Figure 4B), which corresponded to an inflection point at the maximum likelihood observed across all values of K tested. Pairwise F_ST_ values between these metapopulations averaged 0.2 and ranged from 0.054 to 0.574 (Table 2), all of which were significant following Bonferroni correction (p<0.0008). The variance explained by these 11 metapopulations (Fst = 0.09, p = 0.001) was similar to that explained by population when measured within the three metapopulations identified at K = 3. Hypothetical metapopulations identified at K = 11 were generally robust to alternative modes of analysis. We recovered the same groupings upon excluding data from loci D05 and Pgp17, which had revealed some departures from HWE. Furthermore, although parallel analyses with Geneland indicated that the posterior probability was highest for K = 9, the groupings identified by this program were identical to those observed with Structure at K = 11 with the following exceptions: JN grouped with NA/KL and ND grouped with BV/BU/OK. The hierarchical relationships described above were also evident in a NJ tree based on Cavalli-Sforza chord distances (Figure 5).

**Table 2:** Microsatellite-based F_ST_ values for pairwise comparisons among population clusters produced by STRUCTURE at K = 11.

	KU	AR-MY	AP-DK-OG-PD	BG-BK-BN-MK-OS	KB	KK	MF	MS	JN	KL-NA	BU-BV-OK
AR-MY	0.233										
AP-DK-OG-PD	0.277	0.054									
BG-BK-BN-MK-OS	0.329	0.111	0.087								
KB	0.352	0.129	0.164	0.195							
KK	0.444	0.200	0.250	0.270	0.107						
MF	0.331	0.085	0.130	0.169	0.049	0.150					
MS	0.418	0.156	0.181	0.215	0.102	0.166	0.093				
JN	0.448	0.217	0.240	0.282	0.206	0.287	0.117	0.277			
KL-NA	0.395	0.212	0.224	0.263	0.221	0.319	0.136	0.275	0.088		
BU-BV-OK	0.366	0.194	0.217	0.236	0.173	0.253	0.114	0.244	0.098	0.075	
ND	0.574	0.317	0.363	0.366	0.292	0.343	0.262	0.371	0.290	0.262	0.167

All comparisons were significant at a Bonferroni corrected p<0.0008. Individuals from MF exhibited strongly mixed ancestry and were treated as a unique cluster for comparisons of F_ST_.

**Figure 6:**
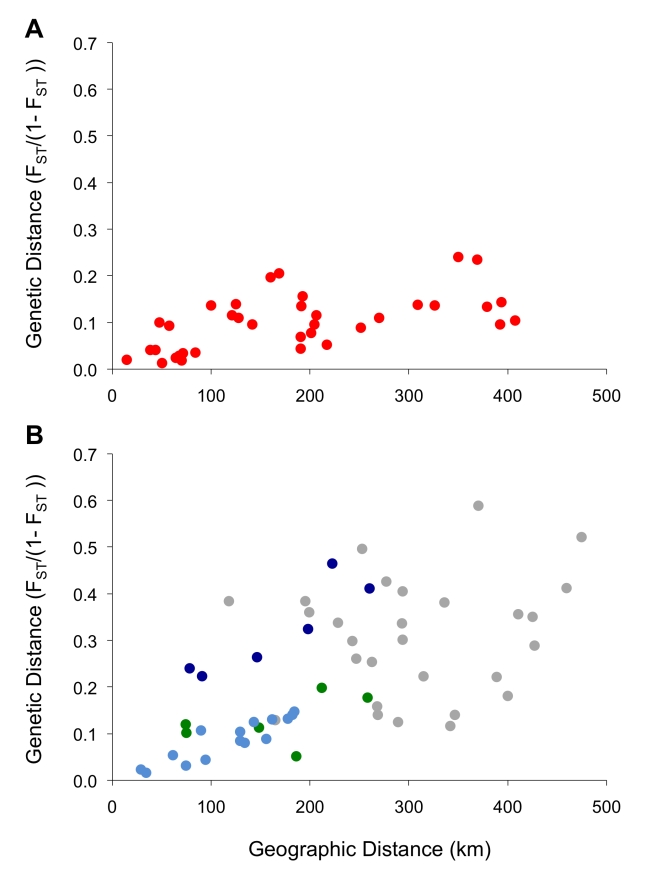
Isolation by distance. Genetic distance (F_ST_/(1- F_ST_)) versus geographic distance is plotted for all pairwise comparisons among populations of *G. f. fuscipes* in northern (A) or southern (B) Uganda. In the lower panel, colored dots represent pairwise comparisons involving only western populations (green) or only southeastern populations (blue). In southeastern Uganda, comparisons involving the population from Ndere Island (ND; dark blue) reveal a discontinuity in the equilibrium between gene flow and drift.

Despite the structure observed above, tests of isolation by distance revealed regional equilibrium between gene flow and genetic drift [45], but this signal was stronger in microsatellites than in mtDNA. For mtDNA, a significant signal of IBD was evident only in the north and only when accounting for the evolutionary signal present in sequence data using Φ_ST_ (R^2^ = 0.23, p = 0.014; Table 3). Here, geographical proximity explained only a relatively small proportion of the overall variance in genetic differentiation between populations. In the south, we did not detect IBD among maternal lineages, suggesting that for female tsetse, drift is a strong force relative to gene flow. Relationships contributing to this lack of signal included, for example, the small pairwise F_ST_ observed between the distantly located populations Kabunkanga (KB) and Okame (OK) and the large pairwise F_ST_ observed between neighboring populations Ndere (ND) and Busime (BU; Table S6). For microsatellites, on the other hand, we detected a significant pattern of IBD in both the north and the south (Table 3) and this was evident in scatterplots of genetic distance versus geographic distance (Figure 6). This association was particularly strong when assessed within the southeastern populations alone (R^2^ = 0.52, p = 0.001; Table 3).

**Table 3:** Correlation statistics for tests of isolation by distance using mitochondrial (mtDNA) and nuclear markers (msats).

Marker	Group	Genetic Distance	R^2^	p	Slope	Intercept	Populations Included
mtDNA	North	F_ST_/(1- F_ST_)	0.10	0.071	0.0075	−0.62	AP, AR, BG, DK, MK, MY, OG, PD, MF
		Φ_ST_/(1- Φ_ST_)	0.23	**0.014**	0.0225	−2.19	AP, AR, BG, DK, MK, MY, OG, PD, MF
	South	F_ST_/(1- F_ST_)	0.04	0.249	0.0116	−1.37	BU, BV, KB, KK, NA, ND, OK
		Φ_ST_/(1- Φ_ST_)	0.07	0.183	0.0147	−1.59	BU, BV, KB, KK, NA, ND, OK
msats	North	F_ST_/(1- F_ST_)	0.34	**0.005**	0.0005	0.0085	AP, AR, BK, BN, DK, MK, MY, OG, OS
	South (K = 2)	F_ST_/(1- F_ST_)	0.35	**0.001**	0.0013	−0.0506	BU, BV, JN, KB, KL, KK, MF, MS, NA, ND, OK
	Southeast (K = 3)	F_ST_/(1- F_ST_)	0.52	**0.001**	0.0021	−0.1170	BU, BV, JN, KL, NA, ND, OK
	West (K = 3)	F_ST_/(1- F_ST_)	0.24	0.123	0.0007	0.0136	KB, KK, MF, MS

Population abbreviations correspond to localities identified in Figure 1 and Figure 4. Significant correlations are highlighted in bold.

Given that two of the populations in the southeast were located on islands (Buvuma (BV) and Ndere (ND)), we tested whether open water presented a barrier to gene flow beyond that imposed by distance alone. A partial Mantel test implemented in IBDWS revealed a significant effect of island isolation on genetic distance, after controlling for geographical distance (r = 0.66, p = 0.023); however, this effect could be explained by the isolation of ND alone. Examining the effect of ND only, a second partial Mantel test revealed a strong effect of island isolation after controlling for geographical distance (r = 0.94, p = 0.001) and also confirmed the strong relationship between genetic distance and geographical distance in the southeast, after controlling for the effect of ND (r = 0.88, p = 0.001). Partial Mantel tests involving BV alone provided no evidence for significant isolation of this population beyond that attributable to geographical distance.

### Population Size and Stability

Bayesian estimates of N_e_ based on single point samples provided evidence for relatively large historical effective population sizes in Busime (BU) in southeastern Uganda, and also in several populations from western and northwestern Uganda: Kabunkanga (KB), Kakoga (KK), Murchison Falls (MF) and Moyo (MY; Table 4). Based on the linkage disequilibrium method, only four populations (BN, BU, MS and ND) exhibited 95% confidence intervals that excluded infinity. Of these, only Bunghazi (BN) and Masindi (MS) also exhibited relatively low effective population sizes based on Bayesian estimates, and these estimates were not influenced by the choice of prior. The population from Ndere Island (ND) was not evaluated via the Bayesian method due to monomorphism at 4 loci.

**Table 4:** Estimates of effective population size (N_e_) calculated with Bayesian and linkage disequilibrium methods.

Population	Bayesian a			Linkage Disequilibrium		
	N_e_	Low	High	N_e_	Low	High
AP	267	151	1029	∞	355	∞
AR	138	80	570	∞	1208	∞
BG^b^	--	--	--	--	--	--
BK	184	101	731	∞	100	∞
BN	153	95	523	32	21	56
BU	6648*	1532	194991	91	43	801
BV	286	129	1259	2499	90	∞
DK	345	179	1490	405	114	∞
JN	239	127	1130	∞	103	∞
KB	1222*	414	9276	244	85	∞
KK	447*	209	3777	162	64	∞
KL	255*	135	1354	105	38	∞
MF	1457	608	10194	∞	129	∞
MK	107	57	470	1001	66	∞
MS	64	36	213	64	37	161
MY	1371	581	9806	∞	228	∞
NA	56	34	230	∞	51	∞
ND^c^	--	--	--	15	7	31
OG	511*	248	2488	∞	188	∞
OK	222*	104	928	517	75	∞
OS	54	34	160	83	36	∞
PD^b^	--	--	--	--	--	--

a Estimates were generated using a prior of 10,000 on N_e_. Estimates that were not robust to changes in prior (*) are indicative of relatively large population sizes.

b Estimates of Ne were not performed for BG or PD due to small sample size.

c Only 9 loci were polymorphic for ND and therefore, Bayesian estimates were not generated.

Detection of recent population bottlenecks was strongly influenced by the model chosen for analysis. After Bonferroni correction (p<0.0024), five populations exhibited significant signatures of a population bottleneck under the IAM model, but only one of these (Okame; OK) also tested positive for a bottleneck under the TPM model (Table 5).

**Table 5:** Significance of tests for population bottlenecks assessed using a Wilcoxon test under an infinite allele (IAM) or two-phase (TPM) model of microsatellite evolution.

Population	p (IAM)	p (TPM)
AP	0.0026	0.1367
AR	**0.0002**	0.3178
BK	0.0040	0.4548
BN	**0.0002**	0.0737
BU	0.2593	0.8669
BV	0.3178	0.8121
DK	0.0239	0.1698
JN	0.0549	0.2593
KB	0.0034	0.4197
KK	0.1527	0.7929
KL	0.1602	0.4155
KU	0.4251	0.6890
MF	0.3934	0.9957
MK	0.1167	0.3110
MS	**0.0001**	0.0026
MY	0.1527	0.8302
NA	**0.0017**	0.0081
ND	0.0645	0.3672
OG	0.2274	0.7929
OK	**0.0002**	**0.0012**
OS	0.0955	0.6323

Tests that remained significant after Bonferroni correction (p = 0.0024) are highlighted in bold.

## Discussion

Genetic structure in both mtDNA and microsatellites confirmed the presence of two distinct lineages of *G. f. fuscipes* in Uganda [12]. This genetic differentiation, if associated with differences in physiology, behavior, and/or symbiont composition affecting vector competence [46]–[49], could have important implications for the epidemiology of trypanosomiasis, as well as vector control. However, the deep structure of *G. f. fuscipes* in Uganda appears to reflect an ancient event that divided northern and southern lineages, and in light of evidence for modern gene flow, is likely to be impermanent.

The isolation of northern and southern lineages, which appear to have diverged on the order of several hundreds of thousands of years before present, may have been facilitated by habitat fragmentation during extreme drought cycles in East Africa that ended only 70,000 years ago [50]. Following the Last Glacial Maximum approximately 20,000 years ago [51], a period when Lake Victoria may have been totally dry [52], increasingly moist conditions would have favored the expansion of the riverine species *G. f. fuscipes* from dry period refugia. Given the perfect latitudinal partitioning of mitochondrial groups, the two divergent lineages likely expanded into their present distribution via distinct pathways. These paths may have split to the north and south of the Blue and Rwenzori mountain ranges, both of which form a barrier to direct colonization from the west. As evidenced by the distribution of mtDNA haplotypes, these two groups currently meet along a zone of contact extending from Bunghazi (BN) in eastern Uganda to Masindi (MS) in western Uganda. This zone of contact falls to the south of Lake Kyoga, which was formerly proposed as a barrier to gene flow between northern and southern flies [12]. Given the absence of obvious alternative geographical barriers to gene flow and the fairly narrow zone of contact, we speculate that northern and southern flies have come into contact only recently.

At present, evidence for admixture between the northern and southern flies is not uniform along the zone of contact. In Bunghazi (BN), we did not detect any deviation from HWE and almost all flies exhibited a mix of southern and northern ancestry, irrespective of their maternal lineage. In this portion of the contact zone, therefore, no barriers to mating are evident between northern and southern lineages. Given the hybrid dysgenesis observed among cryptic taxa within *G. palpalis palpalis* [53], this observation warrants experimental confirmation. Elsewhere in the contact zone, flies exhibited genetic signatures consistent with the introgression of a northern mtDNA haplotype into a nuclear background that allied almost exclusively with either southeastern (JN) or western (MS) flies. This scenario is consistent with rare female-biased dispersal into the contact zone from the north and chance amplification of that northern haplotype by drift in a small effective female population. *Wolbachia*-induced mating incompatibility could also have played a role in driving the northern lineage to relatively high frequency, a possibility currently being investigated. In Junda (JN) and Masindi (MS), the homogeneous nuclear background did not support admixture of northern and southern flies. However, just north of the contact zone, flies captured along the Nile River at Murchison Falls (MF) and along Lake Kyoga at Bugondo (BG) exhibited strong signals of mixed ancestry, confirming our observation in Bunghazi that gene flow between northern and southern lineages is possible and ongoing.

Given the evidence of admixture highlighted above, the genetic differentiation between northern and southern lineages may simply represent a signature of historical allopatric fragmentation that has little bearing on the current movement of genes. However, Bayesian assignment probabilities (Figure 4), which provide an estimate of an individual's recent ancestry, suggested that the directionality of gene flow is constrained, both across the zone of contact and on a wider scale. Importantly, individuals to the south of the zone of contact in southeastern Uganda (blue) exhibited negligible recent shared ancestry with either flies just to the north, or even with flies from western Uganda, some of which possessed identical mtDNA lineages. This is indicative of little modern gene flow from the north or west into the southeast and lends support for PATTEC's choice of the Lake Victoria region as a suitably isolated target for tsetse intervention [6]. In contrast, individuals in the north and west tended to exhibit traces of ancestry from populations lying to the south or southeast, suggestive of a north or northwestern bias to gene flow. We hypothesize that this bias in direction may be linked to passive dispersal of pupae via seasonally flooded river systems, such as the Nile, Semliki and Achwa, all of which follow a north or northwesterly course in Uganda. In support of this mechanism, experimental tests have shown that pupae of both *G. tachinoides* and *G. submorsitans*, can survive for periods of at least 24 hours while submerged in water or saturated soil [54]. Water-borne dispersal, unlike the bidirectional volant dispersal of adult tsetse through habitat adjacent to rivers and lakes, may constrain the direction of movement, but may also allow *G. f. fuscipes* to disperse in the absence of contiguous tracts of favorable habitat. This warrants consideration when devising vector control strategies.

Whatever the dominant mode of dispersal, analyses of isolation by distance using microsatellites reflected an equilibrium between gene flow and drift in both northern and southern regions. In northern Uganda, the slope of the regression of genetic distance on geographic distance was shallow relative to that observed in southern Uganda, suggesting that the homogenizing influence of gene flow is relatively stronger in the north versus the south. The relatively low genetic divergences observed over large geographic distances may have also been influenced by a recent history of sequential founder events occurring from west to east across northern Uganda, a process that is supported by the significant decline in allelic diversity with longitude across the north. Both scenarios emphasize the vagility of *G. f. fuscipes*, albeit at different time scales. In southern Uganda, the slope of the regression of genetic distance on geographic distance was steeper, and at large geographic distances, was driven by the major genetic discontinuity between flies on either side of the gulf in *G. f. fuscipes*' predicted range (between Kabunkanga (KB) and Nkumba (NA); Figure 1). Although mtDNA signatures reflect a historical connection between western and southeastern populations, currently, there appears to be little gene flow and these populations warrant separate consideration. In western Uganda, IBD was not apparent, perhaps owing to the low sample size, or perhaps owing to the fact that nearest-neighbor geographical distances did not capture actual dispersal distances between sites (e.g., measured along riverine corridors). In southeastern Uganda, on the other hand, the signal of IBD was relatively strong, suggesting that ongoing exchange of genetic material is moderating the random allelic variation that would otherwise accumulate in isolated populations undergoing genetic drift alone. The exception was the population from Ndere Island (ND), which was more differentiated than expected based on the pattern of IBD observed in neighboring populations, perhaps due to its isolation from the mainland or its small effective population size. The latter may be the critical factor since Buvuma Island (BV) did not exhibit the same discontinuity. For Buvuma Island, the open water separating the island from the mainland appeared to be no more of a barrier to dispersal than the habitat separating neighboring mainland populations.

The signal of gene flow obtained from IBD analysis is consistent with estimates of dispersal rates for riverine tsetse, which are on the order of tens of kilometers per year [55]–[57]. Reflecting this dispersal capacity, at the smallest scale of analysis, individual populations of *G. f. fuscipes* in Uganda appeared to be genetically homogeneous over the 1–5 km^2^ trapping area that formed our fundamental sampling unit. Although we detected significant deviations from HWE for two loci in a handful of these populations, we did not observe consistent trends in F_IS_ that would provide evidence for any finer scale substructuring of tsetse populations, such as that observed in *G. palpalis*, a related species of riverine tsetse [58]. Extending beyond the immediate trapping locality, we observed significant but relatively small differentiation (F_ST_ <∼0.1) between most populations separated by less than 100 km. This level of differentiation is similar to the differentiation observed among populations of *G. palpalis* at similar scales in Burkina Faso and Equatorial Guinea [59]–[63]. Several of these studies have focused on the isolation implied by this differentiation, but with rare exception [57], absolute values of genetic isolation have yet to be reconciled with actual dispersal rates or the outcomes of vector control. Future efforts should focus on calibrating the consequences of genetic differentiation, perhaps by measuring the rate of reinfestation following eradication of the many tsetse populations for which genetic isolation indices now exist.

Previous observation of significant F_ST_ values among populations of *G. f. fuscipes* in Uganda prompted the conclusion that genetic drift is a much stronger force than gene flow and that perhaps dispersal tendencies have been overestimated [12],[13]. In this study, high F_ST_ values and little evidence for IBD in mtDNA provided some support for this conclusion, at least among females; however, for microsatellite data, IBD analyses were in accord with higher levels of current gene flow. Even if our microsatellite-based estimates of F_ST_ are deemed to be high, modeling has shown that high F_ST_ may persist in the face of high gene flow if environmental heterogeneity contributes to a large variance in the size of individual populations [64],[65]. Our estimates of N_e_ for *G. f. fuscipes* in Uganda, though subject to large confidence intervals, were variable across populations, indicating that this condition may be met. Interestingly though, with the exception of Okame (OK), a site that has experienced continuous trapping of tsetse over the last several years, we did not find strong evidence for bottlenecks in the majority of populations considered. Our power to detect bottlenecks was likely low, since the transient signal of reduced allelic diversity that is indicative of a bottleneck, is unlikely to persist across multiple demographic cycles in populations with historically low N_e_ [66]. At several sites to which we made repeat visits, we observed large changes in trapping rates over the course of a year (unpubl. data), which were presumably linked to changes in water availability and habitat composition. Therefore, it remains possible that severe dry season demographic contractions and attendant high levels of genetic drift could be muting stronger signals of gene flow [65]. Strategies for control of *G. f. fuscipes* in Uganda should account for the movement of tsetse consistent with this gene flow.

In conclusion, implied levels of gene flow among populations of *G. f. fuscipes* in Uganda appear to be consistent with high dispersal capacity, though confirmation of this will require the coordination of genetic studies with mark-recapture experiments. Interestingly, open water did not appear to present an unusual barrier to gene flow and we speculate that rivers may serve as a conduit for passive dispersal. Contrary to hypotheses invoking modern geographical barriers, the largest scale genetic structure apparent in Ugandan populations of *G. f. fuscipes* appears to have arisen from an ancient event that divided northern and southern lineages. Evidence for admixture between these lineages suggests that this structure may be impermanent but future studies should explore the viability of hybrid flies, particularly in light of the northerly-biased gene flow evident across the zone of contact.

## Supporting Information

Table S1 Mitochondrial haplotype information, including frequencies observed across populations and associated GenBank accession numbers.
(0.09 MB DOC)

Table S2 Primer sequences and original source of loci used in this study.
(0.06 MB DOC)

Table S3 Estimates of FIS at 13 microsatellite loci for populations of *G. f. fuscipes*. Significance was assessed at p<0.05 (*) and, after Bonferonni correction by locus, at p<0.002 (**).
(0.09 MB DOC)

Table S4 Allele frequencies observed across populations of *G. fuscipes* at each locus.
(0.07 MB XLS)

Table S5 Microsatellite-based FST values (below diagonal) and significance (above diagonal) for all pairwise comparisons between populations of *G. f. fuscipes* in Uganda, Kenya (ND), Sudan (KU) and the Democratic Republic of Congo (LR; *G.f. quanzensis*).
(0.11 MB DOC)

Table S6 Mitochondrial-based FST values (below diagonal) and significance (above diagonal) for all pairwise comparisons between populations in Uganda, Kenya (ND) and Sudan (KU).
(0.10 MB DOC)
