# Supplementary material for: Phylogeography and Population Structure of Glossina fuscipes fuscipes in Uganda: Implications for Control of Tsetse
Source: PLoS Negl Trop Dis. 2010 Mar 16;4(3):e636. doi: 10.1371/journal.pntd.0000636 (PMC2838784; doi:10.1371/journal.pntd.0000636)
Supplement: Table S1 — Mitochondrial haplotype information, including frequencies observed across populations and associated GenBank accession numbers. (0.09 MB DOC) [file pntd.0000636.s001.doc]

Table S1. Mitochondrial haplotype information, including frequencies observed across populations and associated GenBank accession numbers.

| Haplotype ID | Population | Frequency | GenBank no. |
| --- | --- | --- | --- |
| 1 | KZ | 1 | GU296746 |
| 2 | KK | 1 | GU296747 |
| 3 | KK | 3 | GU296748 |
| 4 | NA | 6 | GU296749 |
| 5 | KZ | 4 | GU296750 |
| 6 | KK | 1 | GU296751 |
| 7 | KZ | 1 | GU296752 |
| 7 | KK | 10 | GU296752 |
| 8 | BV | 2 | GU296753 |
| 9 | BV | 4 | GU296754 |
| 10 | MS | 4 | GU296755 |
| 11 | BV | 2 | GU296756 |
| 12 | JN | 2 | GU296757 |
| 13 | KB | 1 | GU296758 |
| 14 | KB | 1 | GU296759 |
| 15 | BU | 8 | GU296760 |
| 16 | OK | 1 | GU296761 |
| 17 | ND | 15 | GU296762 |
| 18 | OK | 2 | GU296763 |
| 19 | BN | 4 | GU296764 |
| 19 | BU | 7 | GU296764 |
| 19 | BV | 7 | GU296764 |
| 19 | KB | 13 | GU296764 |
| 19 | NA | 9 | GU296764 |
| 19 | OK | 12 | GU296764 |
| 20 | OG | 1 | GU296765 |
| 21 | BG | 5 | GU296766 |
| 21 | BN | 3 | GU296766 |
| 21 | MK | 7 | GU296766 |
| 21 | DK | 3 | GU296766 |
| 22 | OG | 1 | GU296767 |
| 23 | AP | 2 | GU296768 |
| 23 | OG | 1 | GU296768 |
| 24 | AP | 1 | GU296769 |
| 25 | KT | 1 | GU296770 |
| 26 | AP | 1 | GU296771 |
| 26 | OG | 1 | GU296771 |
| 27 | AP | 7 | GU296772 |
| 27 | BG | 2 | GU296772 |
| 27 | BN | 8 | GU296772 |
| 27 | MK | 8 | GU296772 |
| 27 | OG | 5 | GU296772 |
| 27 | PD | 9 | GU296772 |
| 27 | DK | 10 | GU296772 |
| 28 | KU | 1 | GU296773 |
| 29 | KU | 2 | GU296774 |
| 30 | KU | 12 | GU296775 |
| 30 | KT | 4 | GU296775 |
| 30 | MY | 2 | GU296775 |
| 31 | AP | 1 | GU296776 |
| 32 | MS | 11 | GU296777 |
| 33 | AR | 2 | GU296778 |
| 33 | MY | 4 | GU296778 |
| 34 | AR | 3 | GU296779 |
| 35 | AR | 1 | GU296780 |
| 36 | AP | 3 | GU296781 |
| 36 | AR | 9 | GU296781 |
| 36 | MF | 1 | GU296781 |
| 36 | MY | 9 | GU296781 |
| 36 | OG | 1 | GU296781 |
| 36 | PD | 1 | GU296781 |
| 36 | DK | 2 | GU296781 |
| 37 | BG | 6 | GU296782 |
| 37 | JN | 13 | GU296782 |
| 37 | MF | 13 | GU296782 |
| 38 | MF | 1 | GU296783 |
| 39 | LR | 2 | GU296784 |
| 40 | LR | 7 | GU296785 |
| 41 | LR | 1 | GU296786 |
