# Supplementary material for: Phylogeography and Population Structure of Glossina fuscipes fuscipes in Uganda: Implications for Control of Tsetse
Source: PLoS Negl Trop Dis. 2010 Mar 16;4(3):e636. doi: 10.1371/journal.pntd.0000636 (PMC2838784; doi:10.1371/journal.pntd.0000636)
Supplement: Table S2 — Primer sequences and original source of loci used in this study. (0.06 MB DOC) [file pntd.0000636.s002.doc]

Table S2. Primer sequences and original source of loci used in this study.

| Locus | Primer | Primer sequence | Source |
| --- | --- | --- | --- |
| A03 | A03bF | AGGAATCAGTTAGTCTTCTGC | Brown et al. 2008 |
|  | A03bR | ACTCGACCTCATCTATTCTG |  |
| B05 | B05F | CGCGCTTAGCTAGGAAACTC | Abila et al. 2008 |
|  | B05R | AACGATTTGCTGTCCTCGAT |  |
| C07 | C07Fm13b | *GAATTTTAACAAATTGGACTTACAGA | This study |
|  | C07Rb | CCAGGTTAAAACCAGTAACTTCC |  |
| D05 | D05F | TTTCCTTCCAGACGAACTG | Abila et al. 2008 |
|  | D05R | CTTGGTATGGTCGTACATGG |  |
| D101 | D101F | TGCCTTTACACTGCATACTACC | Abila et al. 2008 |
|  | D101R | AAAAAGAGGAGCAATGATGTG |  |
| Gmm8 | Gmm8Fm13 | *CGCGCTTCAATGTTTGCTTTC | Baker et al. 2001 |
|  | Gmm8R | TGCAGATGCAATGCGGAGAG |  |
| GmsCAG29B | GmsCAG29BFm13 | *AACTATTGCTGGGCTCAC | Baker et al. 2001 |
|  | GmsCAG29BRgff | AATTTCACTTCCACTCACCG |  |
| GpB20b | GpB20bFgffm13 | *CAAAAGGGGAAAAGAAAGAAAGAGT | Ouma et al. 2003 |
|  | GPB20bR2 | GTTTCGGCAGTAGATGGCAA |  |
| GpCAG133 | GpCAG133F | ATTTTTGCGTCAACGTGA | Baker et al. 2001 |
|  | GpCAG133R | ATGAGGATGTTGTCCAGTTT |  |
| GpC5b | GpC5bFgffm13 | *GGATATGCGAACCTATACGC | Ouma et al. 2003 |
|  | GpC5bR | CAAGGGTGTGTCGTCTTC |  |
| GpC10b | GpC10bFgffm13 | *TTCAAGCACACTATTGCCAC | Ouma et al. 2003 |
|  | GpC10bR | GCTGGCAAAGAAACTATTGA |  |
| Pgp17 | Pgp17Fm13 | *TGGCAAACTCTTCCATGTTT | Luna et al. 2001 |
|  | Pgp17Rgff | GCTTACGTGAATCGTATCGAAT |  |
| Pgp28 | Pgp28Fm13 | *TCAAATTGTTCCCATCAAGGA | Luna et al. 2001 |
|  | Pgp28R | ATCGTTTTTAAAGGGTTTTAAGTTT |  |

* indicates the addition of an m13 tail (tcccagtcacgacgt) to the 5’ end of the primer.
