# Supplementary material for: Phylogeography and Population Structure of Glossina fuscipes fuscipes in Uganda: Implications for Control of Tsetse
Source: PLoS Negl Trop Dis. 2010 Mar 16;4(3):e636. doi: 10.1371/journal.pntd.0000636 (PMC2838784; doi:10.1371/journal.pntd.0000636)
Supplement: Table S3 — Estimates of FIS at 13 microsatellite loci for populations of G. f. fuscipes. Significance was assessed at p<0.05 (*) and, after Bonferonni correction by locus, at p<0.002 (**). (0.09 MB DOC) [file pntd.0000636.s003.doc]

Table S3. Estimates of F_IS_ at 13 microsatellite loci for populations of *G. f. fuscipes*. Significance was assessed at p < 0.05 (*) and, after Bonferonni correction by locus, at p < 0.002 (**).

| Population | Microsatellite Locus | | | | | | | | | | | | |
| --- | --- | --- | --- | --- | --- | --- | --- | --- | --- | --- | --- | --- | --- |
| Locality | A03b | B05 | B20b | CAG29 | C5b | C7b | D05 | D101 | Gmm8 | GpCAG133 | GpC10 | Pgp17 | Pgp28 |
| **UGANDA** |  |  |  |  |  |  |  |  |  |  |  |  |  |
| AP | 0.178* | 0.184 | -0.016 | 0.098 | -0.011 | 0.086 | 0.07 | -0.057 | -0.003 | -0.145 | 0.096 | 0.392** | -0.032 |
| AR | -0.051 | 0.318* | -0.019 | 0.036 | -0.055 | 0.025 | 0.11 | -0.227 | -0.306 | 0 | 0.002 | 0.139 | -0.014 |
| BG | -0.06 | 0.104 | -0.176 | -0.103 | NA | -0.012 | 0.044 | -0.043 | 0.065 | -0.244 | 0.123 | -0.043 | 0.19 |
| BK | -0.008 | 0.038 | -0.091 | 0 | NA | -0.124 | 0.301** | -0.164 | -0.008 | -0.03 | 0.021 | 0.158 | -0.016 |
| BN | 0.248 | 0.077 | 0.19 | NA | NA | 0.041 | 0.099 | -0.054 | 0.062 | 0.076 | 0.171 | 0.043 | -0.044 |
| BU | -0.02 | 0.062 | 0.019 | 0.483 | NA | 0.115 | 0.061 | -0.031 | 0.235 | 0.128 | 0.274 | 0.046 | -0.084 |
| BV | -0.049 | -0.188 | 0.061 | 0 | -0.013 | 0.036 | 0.237 | 0.182 | 0.064 | -0.087 | 0.046 | 0.03 | 0.034 |
| DK | 0.079 | 0.215 | -0.047 | 0.177 | 0.073 | 0.029 | 0.116 | -0.033 | 0.16 | 0.016 | 0.012 | 0.413** | 0.086 |
| JN | 0.018 | 0.158 | -0.084 | 0 | NA | -0.036 | 0.214 | -0.054 | -0.018 | -0.013 | 0.112 | -0.064 | 0.005 |
| KB | -0.008 | -0.023 | -0.054 | 0.228 | 0.132 | -0.065 | -0.026 | -0.068 | -0.24* | -0.068 | 0.181 | 0.267** | 0.077 |
| KK | 0.1 | -0.049 | 0.111 | 0.088 | 0.062 | -0.235 | 0.533** | -0.074 | 0.09 | -0.033 | -0.047 | 0.226** | 0.243 |
| KL | 0.083 | -0.031 | 0.006 | NA | NA | -0.061 | 0.002 | -0.156 | 0.664* | -0.041 | 0.136 | -0.011 | -0.066 |
| MF | 0.086 | 0.076 | 0.034 | -0.015 | 0.164 | 0.131 | 0.167 | 0.096 | -0.106* | -0.039 | -0.093 | 0.033 | 0.083 |
| MK | 0.024 | -0.001 | 0.062 | 0.009 | NA | 0.032 | -0.009 | 0 | 0.016 | -0.175 | -0.059 | 0.095 | -0.052 |
| MS | 0.068 | -0.161 | 0.006 | -0.052 | -0.081 | 0.176 | 0.129 | 0.037 | -0.158 | -0.068 | 0.02 | 0.063 | -0.021 |
| MY | 0.039 | 0.123 | 0.073 | -0.074 | -0.046 | 0.047 | -0.003 | -0.042 | 0.12 | -0.12 | -0.056 | 0.04 | 0.083* |
| NA | 0.01 | -0.233 | -0.102 | -0.014 | NA | -0.151 | -0.062 | -0.097 | NA | -0.023 | -0.176 | -0.061 | 0.087 |
| ND (Kenya) | -0.052 | -0.114 | 0.077 | NA | NA | -0.079 | -0.174 | NA | 0.147 | 0.455* | -0.085 | NA | -0.093 |
| OG | -0.133 | 0.133* | -0.023 | 0.101 | 0.177 | -0.064 | -0.02 | 0.234 | 0.048 | -0.104 | 0.045 | 0.063 | -0.006 |
| OK | 0.017 | 0.08 | -0.053 | NA | NA | -0.093 | 0.409 | 0.101 | -0.23 | -0.021 | 0.003 | 0.119 | -0.071 |
| OS | 0.146 | 0.435* | 0.144 | -0.069 | 0 | 0.011 | -0.046 | 0.279 | -0.064 | -0.027 | -0.016 | -0.029 | 0.003 |
| PD | 0.044 | 0.125 | 0.06 | -0.103 | -0.091 | -0.217 | -0.303 | -0.091 | -0.347 | 0.154 | -0.096 | 0.064 | 0.15 |
| Pooled^a^ | 0.168** | 0.295** | 0.14** | 0.219** | 0.202** | 0.228** | 0.265** | 0.364** | 0.254** | 0.166** | 0.215** | 0.212** | 0.181** |
| **SUDAN** |  |  |  |  |  |  |  |  |  |  |  |  |  |
| KU | 0.091* | -0.167 | -0.053 | NA | -0.355 | -0.061 | -0.2 | 0.07 | 0.073 | -0.033 | 0.162 | 0.098 | 0.091 |
| **DEM. REP. CONGO** |  |  |  |  |  |  |  |  |  |  |  |  |  |
| LR | 0.13 | -0.04 | 0.016 | 0.089 | 0.052 | -0.093 | -0.047 | -0.034 | -0.076 | -0.365 | 0.283** | 0.113* | 0.232 |

^a^ Includes all samples from Uganda and the adjacent population in Kenya.
