# Supplementary material for: Phylogeography and Population Structure of Glossina fuscipes fuscipes in Uganda: Implications for Control of Tsetse
Source: PLoS Negl Trop Dis. 2010 Mar 16;4(3):e636. doi: 10.1371/journal.pntd.0000636 (PMC2838784; doi:10.1371/journal.pntd.0000636)
Supplement: Table S5 — Microsatellite-based FST values (below diagonal) and significance (above diagonal) for all pairwise comparisons between populations of G. f. fuscipes in Uganda, Kenya (ND), Sudan (KU) and the Democratic Republic of Congo (LR; G.f. quanzensis). (0.11 MB DOC) [file pntd.0000636.s005.doc]

Table S5. Microsatellite-based F_ST_ values (below diagonal) and significance (above diagonal) for all pairwise comparisons between populations of *G. f. fuscipes* in Uganda, Kenya (ND), Sudan (KU) and the Democratic Republic of Congo (LR; *G.f. quanzensis*).

|  | AP | AR | BG | BK | BN | BU | BV | DK | JN | KB | KK | KL | KU | MF | MK | MS | MY | NA | ND | OG | OK | OS | PD | LR |
| --- | --- | --- | --- | --- | --- | --- | --- | --- | --- | --- | --- | --- | --- | --- | --- | --- | --- | --- | --- | --- | --- | --- | --- | --- |
| AP |  | * | * | * | * | * | * | * | * | * | * | * | * | * | * | * | * | * | * | * | * | * | * | * |
| AR | 0.073 |  | * | * | * | * | * | * | * | * | * | * | * | * | * | * | * | * | * | * | * | * | * | * |
| BG | 0.103 | 0.108 |  | * | * | * | * | * | * | * | * | * | * | * | * | * | * | * | * | * | * | * | * | * |
| BK | 0.136 | 0.126 | 0.117 |  | * | * | * | * | * | * | * | * | * | * | * | * | * | * | * | * | * | * | * | * |
| BN | 0.105 | 0.095 | 0.062 | 0.021 |  | * | * | * | * | * | * | * | * | * | * | * | * | * | * | * | * | * | * | * |
| BU | 0.217 | 0.209 | 0.168 | 0.258 | 0.181 |  | * | * | * | * | * | * | * | * | * | * | * | * | * | * | * | * | * | * |
| BV | 0.221 | 0.201 | 0.186 | 0.263 | 0.191 | 0.032 |  | * | * | * | * | * | * | * | * | * | * | * | * | * | * | * | * | * |
| DK | 0.034 | 0.097 | 0.101 | 0.099 | 0.087 | 0.261 | 0.263 |  | * | * | * | * | * | * | * | * | * | * | * | * | * | * | * | * |
| JN | 0.250 | 0.233 | 0.205 | 0.311 | 0.243 | 0.123 | 0.094 | 0.291 |  | * | * | * | * | * | * | * | * | * | * | * | * | * | * | * |
| KB | 0.173 | 0.121 | 0.110 | 0.200 | 0.143 | 0.182 | 0.183 | 0.190 | 0.206 |  | * | * | * | * | * | * | * | * | * | * | * | * | * | * |
| KK | 0.259 | 0.186 | 0.182 | 0.285 | 0.227 | 0.264 | 0.277 | 0.283 | 0.287 | 0.107 |  | * | * | * | * | * | * | * | * | * | * | * | * | * |
| KL | 0.227 | 0.222 | 0.199 | 0.283 | 0.210 | 0.075 | 0.052 | 0.274 | 0.078 | 0.203 | 0.299 |  | * | * | * | * | * | * | * | * | * | * | * | * |
| KU | 0.279 | 0.247 | 0.373 | 0.337 | 0.328 | 0.389 | 0.376 | 0.317 | 0.448 | 0.352 | 0.444 | 0.406 |  | * | * | * | * | * | * | * | * | * | * | * |
| MF | 0.134 | 0.081 | 0.099 | 0.175 | 0.114 | 0.126 | 0.114 | 0.163 | 0.117 | 0.049 | 0.150 | 0.126 | 0.331 |  | * | * | * | * | * | * | * | * | * | * |
| MK | 0.171 | 0.190 | 0.194 | 0.041 | 0.092 | 0.374 | 0.379 | 0.120 | 0.421 | 0.297 | 0.370 | 0.402 | 0.425 | 0.272 |  | * | * | * | * | * | * | * | * | * |
| MS | 0.190 | 0.148 | 0.150 | 0.229 | 0.183 | 0.252 | 0.253 | 0.213 | 0.277 | 0.102 | 0.166 | 0.265 | 0.418 | 0.093 | 0.299 |  | * | * | * | * | * | * | * | * |
| MY | 0.065 | 0.028 | 0.116 | 0.119 | 0.089 | 0.212 | 0.208 | 0.081 | 0.240 | 0.155 | 0.238 | 0.225 | 0.257 | 0.104 | 0.194 | 0.185 |  | * | * | * | * | * | * | * |
| NA | 0.222 | 0.221 | 0.227 | 0.282 | 0.216 | 0.115 | 0.096 | 0.273 | 0.111 | 0.230 | 0.332 | 0.022 | 0.400 | 0.140 | 0.399 | 0.278 | 0.222 |  | * | * | * | * | * | * |
| ND | 0.395 | 0.330 | 0.365 | 0.413 | 0.345 | 0.194 | 0.210 | 0.426 | 0.290 | 0.292 | 0.343 | 0.245 | 0.574 | 0.262 | 0.527 | 0.371 | 0.373 | 0.318 |  | * | * | * | * | * |
| OG | 0.014 | 0.050 | 0.086 | 0.119 | 0.088 | 0.224 | 0.223 | 0.024 | 0.242 | 0.144 | 0.239 | 0.226 | 0.301 | 0.104 | 0.164 | 0.156 | 0.043 | 0.220 | 0.399 |  | * | * | NS | * |
| OK | 0.215 | 0.189 | 0.147 | 0.248 | 0.171 | 0.016 | 0.043 | 0.254 | 0.116 | 0.154 | 0.225 | 0.082 | 0.385 | 0.107 | 0.367 | 0.232 | 0.201 | 0.128 | 0.183 | 0.214 |  | * | * | * |
| OS | 0.122 | 0.120 | 0.086 | 0.019 | 0.035 | 0.257 | 0.256 | 0.083 | 0.284 | 0.198 | 0.278 | 0.276 | 0.357 | 0.165 | 0.040 | 0.221 | 0.121 | 0.277 | 0.421 | 0.102 | 0.244 |  | * | * |
| PD | 0.022 | 0.043 | 0.095 | 0.121 | 0.080 | 0.219 | 0.208 | 0.043 | 0.244 | 0.158 | 0.254 | 0.215 | 0.314 | 0.110 | 0.190 | 0.194 | 0.036 | 0.212 | 0.432 | 0.004 | 0.209 | 0.106 |  | * |
| LR | 0.177 | 0.159 | 0.205 | 0.254 | 0.219 | 0.279 | 0.273 | 0.210 | 0.318 | 0.231 | 0.307 | 0.295 | 0.318 | 0.196 | 0.320 | 0.271 | 0.191 | 0.284 | 0.454 | 0.178 | 0.275 | 0.229 | 0.154 |  |

All comparisons were significant following sequential Bonferroni correction (*) unless indicated (NS).
