# Supplementary material for: Phylogeography and Population Structure of Glossina fuscipes fuscipes in Uganda: Implications for Control of Tsetse
Source: PLoS Negl Trop Dis. 2010 Mar 16;4(3):e636. doi: 10.1371/journal.pntd.0000636 (PMC2838784; doi:10.1371/journal.pntd.0000636)
Supplement: Table S6 — Mitochondrial-based FST values (below diagonal) and significance (above diagonal) for all pairwise comparisons between populations in Uganda, Kenya (ND) and Sudan (KU). (0.10 MB DOC) [file pntd.0000636.s006.doc]

Table S6. Mitochondrial-based F_ST_ values (below diagonal) and significance (above diagonal) for all pairwise comparisons between populations in Uganda, Kenya (ND) and Sudan (KU).

|  | AP | AR | BG | BN | BU | BV | DK | JN | KB | KK | KT | KU | KZ | MF | MK | MS | MY | NA | ND | OG | OK | PD |
| --- | --- | --- | --- | --- | --- | --- | --- | --- | --- | --- | --- | --- | --- | --- | --- | --- | --- | --- | --- | --- | --- | --- |
| AP |  | * | * | NS | * | * | NS | * | * | * | * | * | * | * | NS | * | * | * | * | NS | * | NS |
| AR | 0.215 |  | * | * | * | * | * | * | * | * | * | * | * | * | * | * | NS | * | * | * | * | * |
| BG | 0.229 | 0.358 |  | NS | * | * | NS | NS | * | * | * | * | * | NS | NS | * | * | * | * | NS | * | * |
| BN | 0.062 | 0.367 | 0.219 |  | * | NS | NS | * | * | * | * | * | * | * | NS | * | * | * | * | NS | * | NS |
| BU | 0.352 | 0.424 | 0.402 | 0.326 |  | NS | * | * | NS | * | * | * | * | * | * | * | * | NS | * | * | NS | * |
| BV | 0.257 | 0.329 | 0.304 | 0.217 | 0.196 |  | * | * | NS | * | * | * | * | * | * | * | * | NS | * | * | NS | * |
| DK | 0.022 | 0.374 | 0.271 | 0.023 | 0.467 | 0.371 |  | * | * | * | * | * | * | * | NS | * | * | * | * | NS | * | NS |
| JN | 0.495 | 0.567 | 0.248 | 0.552 | 0.610 | 0.514 | 0.610 |  | * | * | * | * | * | NS | * | * | * | * | * | * | * | * |
| KB | 0.490 | 0.562 | 0.548 | 0.412 | 0.336 | 0.176 | 0.605 | 0.748 |  | * | * | * | * | * | * | * | * | NS | * | * | NS | * |
| KK | 0.348 | 0.419 | 0.398 | 0.405 | 0.462 | 0.367 | 0.462 | 0.605 | 0.600 |  | * | * | NS | * | * | * | * | * | * | * | * | * |
| KT | 0.366 | 0.454 | 0.428 | 0.436 | 0.510 | 0.389 | 0.510 | 0.709 | 0.702 | 0.504 |  | NS | NS | * | * | * | NS | * | * | NS | * | * |
| KU | 0.438 | 0.510 | 0.492 | 0.495 | 0.552 | 0.457 | 0.552 | 0.695 | 0.691 | 0.548 | -0.052 |  | * | * | * | * | * | * | * | * | * | * |
| KZ | 0.302 | 0.388 | 0.360 | 0.370 | 0.442 | 0.324 | 0.442 | 0.636 | 0.629 | 0.365 | 0.493 | 0.556 |  | * | * | * | * | * | * | NS | * | * |
| MF | 0.484 | 0.544 | 0.239 | 0.548 | 0.605 | 0.510 | 0.601 | -0.014 | 0.743 | 0.600 | 0.702 | 0.691 | 0.629 |  | * | * | * | * | * | * | * | * |
| MK | 0.138 | 0.424 | 0.190 | 0.051 | 0.467 | 0.371 | 0.032 | 0.610 | 0.605 | 0.462 | 0.510 | 0.552 | 0.442 | 0.605 |  | * | * | * | * | NS | * | NS |
| MS | 0.410 | 0.481 | 0.462 | 0.467 | 0.524 | 0.429 | 0.524 | 0.667 | 0.662 | 0.519 | 0.587 | 0.610 | 0.517 | 0.662 | 0.524 |  | * | * | * | * | * | * |
| MY | 0.232 | -0.001 | 0.373 | 0.381 | 0.438 | 0.343 | 0.389 | 0.581 | 0.576 | 0.433 | 0.412 | 0.467 | 0.406 | 0.559 | 0.438 | 0.495 |  | * | * | * | * | * |
| NA | 0.362 | 0.433 | 0.412 | 0.308 | 0.272 | 0.140 | 0.476 | 0.619 | 0.196 | 0.471 | 0.522 | 0.562 | 0.455 | 0.614 | 0.476 | 0.533 | 0.448 |  | * | * | NS | * |
| ND | 0.619 | 0.690 | 0.685 | 0.676 | 0.733 | 0.638 | 0.733 | 0.876 | 0.871 | 0.729 | 0.903 | 0.819 | 0.822 | 0.871 | 0.733 | 0.790 | 0.705 | 0.743 |  | * | * | * |
| OG | -0.059 | 0.264 | 0.221 | 0.032 | 0.357 | 0.251 | 0.003 | 0.522 | 0.516 | 0.351 | 0.371 | 0.455 | 0.299 | 0.513 | 0.115 | 0.422 | 0.281 | 0.367 | 0.672 |  | * | NS |
| OK | 0.438 | 0.510 | 0.492 | 0.358 | 0.286 | 0.134 | 0.552 | 0.695 | -0.009 | 0.548 | 0.627 | 0.638 | 0.556 | 0.690 | 0.552 | 0.610 | 0.524 | 0.158 | 0.819 | 0.455 |  | * |
| PD | 0.113 | 0.535 | 0.475 | 0.158 | 0.609 | 0.505 | 0.034 | 0.772 | 0.766 | 0.604 | 0.731 | 0.706 | 0.642 | 0.765 | 0.268 | 0.673 | 0.551 | 0.620 | 0.920 | 0.095 | 0.706 |  |

Comparisons were significant following sequential Bonferroni correction (*) unless indicated (NS).
